# Effects of fertilization on litter decomposition dynamics and nutrient release in orchard systems

**DOI:** 10.3389/fpls.2024.1467689

**Published:** 2024-12-05

**Authors:** Huayue Nie, Chunhe You, Jixi Gao

**Affiliations:** ^1^ School of Ecology, Environment and Resources, Guangdong University of Technology, Guangzhou, China; ^2^ Satellite Application Center for Ecology and Environment, Ministry of Ecology and Environment, Beijing, China; ^3^ School of Grassland Science, Beijing Forestry University, Beijing, China

**Keywords:** litter decomposition, nutrient additions, element release, nitrogen, phosphorus

## Abstract

Plant litter decomposition is a significant ecosystem function that regulates nutrient cycling, soil fertility, and biomass production. It is heavily regulated by nutrient intake. The effects of exogenous nutrients on litter decomposition are not yet fully understood. To determine how *Eriobotrya japonica* litter decomposition responds to adding nutrients, we used the decomposition litter bag method in the laboratory for 180 days. There were five different nutrient treatment levels were used: control (no addition), low nitrogen addition (LN; 100 kg N·ha^−1^·year^−1^), high nitrogen addition (HN; 200 kg N·ha^−1^·year^−1^), phosphorus addition (P; 50 kg P·ha^−1^·year^−1^), and micronutrient addition (M; 50 kg M·ha^−1^·year^−1^). According to a repeated-measures analysis of variance, adding N reduced the remaining mass (*p* < 0.01) by 4.1% compared to the CK group. In contrast, adding M increased the remaining mass (*p* < 0.01) by 6.8% compared to the CK group. Adding P had no significant effect on the remaining mass. Although the amount of residual carbon (C) was unaffected, adding N increased the level of residual N in the litter. Litter C content, K content, N concentration, and C/N ratio were linearly correlated to the remaining litter (*p* < 0.01). Although adding nutrients decreased soil enzyme activity later in the decomposition process, no significant correlation was detected between enzyme activity and the remaining mass. N fertilization treatments decreased the soil microbial diversity index. The addition of nitrogen and micronutrients reduced the abundance of Acidobacteria, while HN addition increased the abundance of Actinobacteria. The addition of micronutrients increased the abundance of Proteobacteria. These results imply that N-induced alterations in the element content of the litter regulated the effects of nutrient inputs on litter decomposition. This study can be a reference for the fertilization-induced decomposition of agricultural waste litter.

## Introduction

In addition to maintaining ecosystem production and fertilizer application, litter decomposition supplies vital nutrients for plant development and consumption by soil microbes (Aerts et al., 2006; Huangfu and Wei, 2018; Canessa et al., 2021). According to previous studies, the environmental factors (temperature and moisture), initial litter element quality (N content, C content, C/N ratio, and lignin/N ratio), enzyme activities, and the decomposer community of soil microorganisms and fauna all have a significant impact on litter decomposition rate (Diepen et al., 2016; Lucas et al., 2016; Zhou et al., 2017). Nutrient availability is considered a key indicator of the initial stages of litter decomposition (Hobbie, 2005; Van et al., 2013).

The decomposition of litter is suppressed (Zhou et al., 2017; Tao et al., 2019) or accelerated (Downs et al., 1996; Ren et al., 2018) by the addition of N. N fertilizer frequently affects how quickly litter decomposes by altering the soil conditions in which litter decomposes (Gill et al., 2021). N, for example, may alter the activity of soil organisms by enriching fungi and bacteria, increasing the density of soil fauna (Carrera and Bertiller, 2013), or changing their enzyme activity (Song et al., 2018). While phosphorus (P), calcium (Ca), magnesium (Mg), manganese (Mn), and iron have been less frequently evaluated in research on litter decomposition, they are nonetheless closely associated with decomposition rates. For example, P was necessary to supply nutrients to the decomposers in the early stages of decomposition (Van et al., 2016). Due to the effect of Mn on the synthesis and activity of lignin-degrading enzymes, Mn addition had a favorable impact on litter decomposition (Keiluweit et al., 2015). Trum et al. (2015) discovered that Mn enhancement improved the dissolution of dissolved organic C components during degradation by increasing lignin decomposition. Therefore, Mn addition may influence litter decomposition by increasing the fragile substrates available to decomposers (Wu et al., 2022). In summary, the addition of trace elements changes litter decomposition by affecting the decomposers (Berg et al., 2007). It has an ambiguous impact on litter decomposition by increasing microbial diversity and altering the soil microbial community (fungi:bacteria ratio) (Gill et al., 2021).

Nutrient inputs to soil can regulate microbial communities through their influence on the soil resources by providing energy and nutrients or through predation by protists (Asiloglu et al., 2021). It is thought that in nutrient-limited environments, microbial development and activity are constrained by the low availability of C and nutrients. Therefore, the input of nutrients will promote microbial activity (Renella et al., 2006). For example, exogenous N inputs can inhibit phagotrophic protists and thus promote the growth of specific bacterial groups in subtropical ecosystems (Zhao et al., 2020). However, excessive input of nitrogen leads to soil acidification, reducing the specific abundance of ammonia-oxidizing bacteria or overall microbial diversity (Zhong et al., 2016). When P was added, the bacterial diversity and the abundance of *nirK* and *nirS* genes were increased (Wang et al., 2018). However, the influence of fertilization on microbial activity has been inconclusive, with studies reporting increases (Jing et al., 2024; Fu et al., 2023), decreases (Kumar et al., 2018; Morrison et al., 2018), and even no effect (Hobbie and Vitousek, 2000) on microbial abundance with fertilization. Overall, studies have shown that fertilizer inputs can increase the abundance of protists that consume microbes (Delgado-Baquerizo et al., 2017; Guo et al., 2021), but can have negative effects when added excessively.

The effects of nutrient addition on soil enzyme activity and microbial community are well established. In the presence of easily accessible nutrients, microbes reduce the distribution of resources for enzyme synthesis and release (Kumar et al., 2018). The addition of one nutrient can affect not only the activities of the enzymes participating in that specific nutrient cycle but also the activities of other enzymes involved in the cycling of other nutrients. For instance, xylanase activity (engaged in the degradation of hemicellulose) diminished in the presence of mineral nitrogen (Chen et al., 2014). Nutrient addition has an impact on enzyme activity, while soil enzymes are responsible for the biological composition of organic matter (Allison and Vitousek, 2004). Various correlations exist between soil enzyme activity and litter mass reduction (Song et al., 2018). Therefore, additional research on enzyme activity and microbial alterations during decomposition is required to comprehend how litter decomposes in the face of environmental change.

Furthermore, because it is the primary source of agricultural waste, litter management has always been a priority in orchard management. Although N, P, and micronutrients are ubiquitous fertilizers, how they affect litter decomposition is still unknown. As a result, while investigating litter decomposition responses, it is becoming increasingly important to investigate the effects of external fertilizer inputs. We collected fresh leaf litter of *Eriobotrya japonica* from plantations in southern China. We investigated the interaction between the soil microbial community and enzyme activities for 180 days of decomposition in a stable temperature and humidity environment and the influence of exogenous nutrient additions on litter decomposition rates with the decomposition bag method. We hypothesized that adding exogenous nutrients would increase soil enzyme activity and microbial diversity, increasing litter decomposition.

## Methods

### Materials and samples

Litter samples were collected from plantations in Jiangsu Province, southern China (31°2′42″N, 120°19′7″E). This site has a subtropical monsoon marine environment with 1,100 mm of annual mean rainfall and 15.7°C of annual mean air temperature. *E. japonica*, *Citrus reticulata*, and *Pyrus* spp. are the main tree species in this area. In October 2021, fresh *E. japonica* litter samples (intact and unpurified) were collected. **Supplementary Table S1** displays the initial values of the elements found in *E. japonica* litter. The litter was air-dried before being packed into nylon mesh bags (10 cm × 8 cm, with 0.1-cm apertures). Soil samples were collected from an artificial forest at the Chinese Academy of Environmental Sciences, Beijing. The basic chemical properties of the soils are listed in **Supplementary Table S2**.

### Plot design and fertilization

The experiment was run in a greenhouse (25°C–30°C) to reduce the influence of climatic variations on litter decomposition. There were five treatment groups set up: control (CK; no addition), low N addition (LN; 100 kg N·ha^−1^·year^−1^), high N addition (HN; 200 kg N·ha^−1^·year^−1^), P addition (P; 50 kg P·ha^−1^ year^−1^), and micronutrient addition (M; 50 kg M·ha^−1^·year^−1^). The raw materials applied were nitrogen fertilizer (467 kg N·kg^−1^), phosphorus fertilizer (70 kg P·kg^−1^), and agricultural fertilizers (40 kg micronutrients·kg^−1^) rich in calcium, magnesium, iron, and manganese. Five 60 × 50 cm plots were created in the greenhouse at the beginning of November 2021. With 18 duplicate bags, each containing 2 g of litter, evenly distributed over the soil surface, each plot corresponded to a treatment. The soil of each plot was evenly sprayed with 1,000 mL of pure water after the fertilizer had been dissolved in it. The CK treatment was sprayed with 1 L of water without fertilizer. Water was replenished regularly by weighing methods to control the soil moisture content to 60% of the field’s water capacity throughout the experiment.

### Litter sampling and analysis

Three bags from each plot were sampled after 9, 18, 36, 64, 117, and 180 days had passed since decomposition began. Prior to being oven-dried to a constant weight at 60°C and weighed, the remaining litter in the nylon bags was meticulously cleaned of soil and other residues. A percentage of the initial litter mass was used to calculate the remaining litter (%). The dry litter’s total C and N contents were calculated (Zhang et al., 2015) using an elemental analyzer (2400 IICHNS/O, PerkinElmer, Waltham, MA, USA). Total P content was determined colorimetrically after acidified ammonium persulfate digestion (Chen et al., 2013).

### Soil sampling and analysis

The soil from the plots was promptly collected at the end of the experiment. Any remaining plant, root, or gravel components were manually removed from the soil samples. One part of the sample was used to determine enzyme activity, and the other part was used to analyze microbial diversity and composition. Four soil enzyme activities were measured for this study: 1,4-β-glucosidase (BG) for cellulose decomposition, phenol oxidase as a typical ligninolytic oxidoreductase, 1,4-β-*N*-acetylglucosaminidase (NAG) for chitin decomposition, and acid phosphatase (ACP) for the hydrolysis of ester-bonded phosphate and protein. Nitrobenzene-β-d-glucopyranoside, pyrogallol, *p*-nitrophenyl-β-*n*-acetylglucopyranoside, and phenyl-disodium phosphate were the substrates used to determine the enzyme activity (Saiya-Cork et al., 2002). To assess the component of the soil microbial population, phospholipid fatty acid analyses were performed. Soil genomic DNA was taken out following the manufacturer’s instructions using a FastDNA^®^ Spin kit for soil. Purified PCR products were quantified by Qubit^®^3.0 (Life Invitrogen, USA, Alaska), and all 24 amplicons whose barcodes were different were mixed equally. The pooled DNA product was used to construct the Illumina paired-end library following Illumina’s genomic DNA library preparation procedure. Then, the amplicon library was paired-end sequenced (2 × 250) on an Illumina MiSeq platform (Shanghai BIOZERON Co., Ltd., Shanghai, China) according to the standard protocols. Operational taxonomic units (OTUs) were clustered with a 97% similarity cutoff using UPARSE, and chimeric sequences were identified and removed using UCHIME. A rarefaction analysis based on Mothur v.1.21.1 was conducted to reveal the diversity indices, including the Chao, ACE, and Shannon diversity indices (Frostegård et al., 1993).

### Data analysis

The Kolmogorov–Smirnov and Levene’s tests served to determine the normality and homogeneity of variance of the data. The between-group difference in soil enzyme activity and the microbial diversity index at a specific time was confirmed using Dunnett’s *t*-test for multiple comparisons. A multi-factor analysis of variance was performed to determine the effect of nutrient addition on the remaining mass and nutrients in the litter. The relationships between remaining mass (%), remaining nutrients, soil enzyme activity, and microbial diversity indices were analyzed using linear regression. SPSS, Inc., 13.0 was used for the given statistics (Chicago, IL, USA).

## Results

### Effects of nutrient addition on litter decomposition and element content

**Figure 1:**
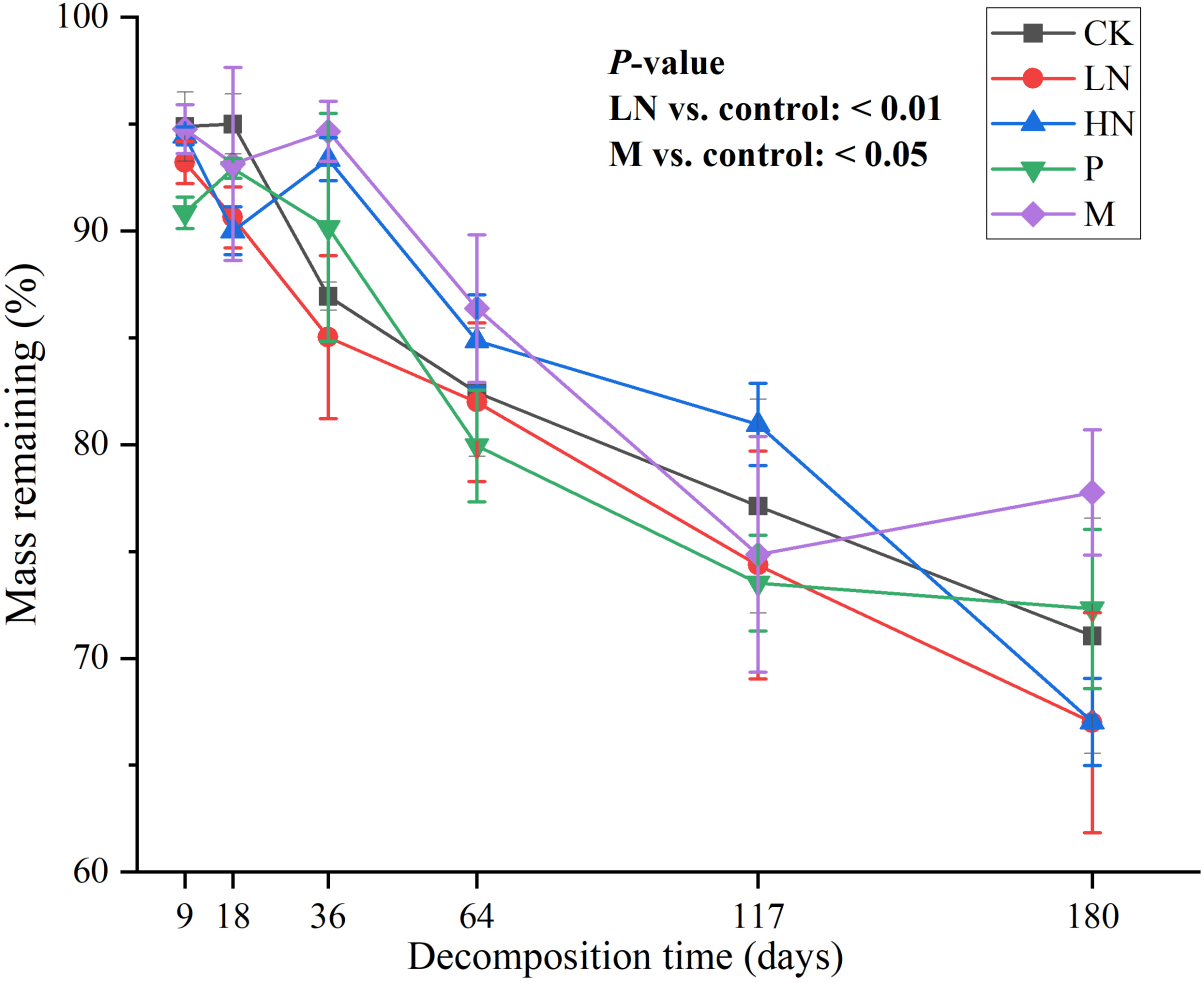
Remaining mass of leaf litter in various nutrient addition treatments. *p* < 0.05 indicates that treatment significantly affects mass loss. CK, control; HN, high concentration N addition; LN, low concentration N addition; P, P addition; M, micronutrient addition.

The two treatment groups with N addition had lower remaining mass than the CK group after 180 days of decomposition. However, during the period of 36–117 days, the remaining mass of the high N addition group was higher than that of the CK group. The M and high N addition groups significantly inhibited and promoted mass loss, respectively (*p* < 0.05) (**Figure 1**).

**Figure 2:**
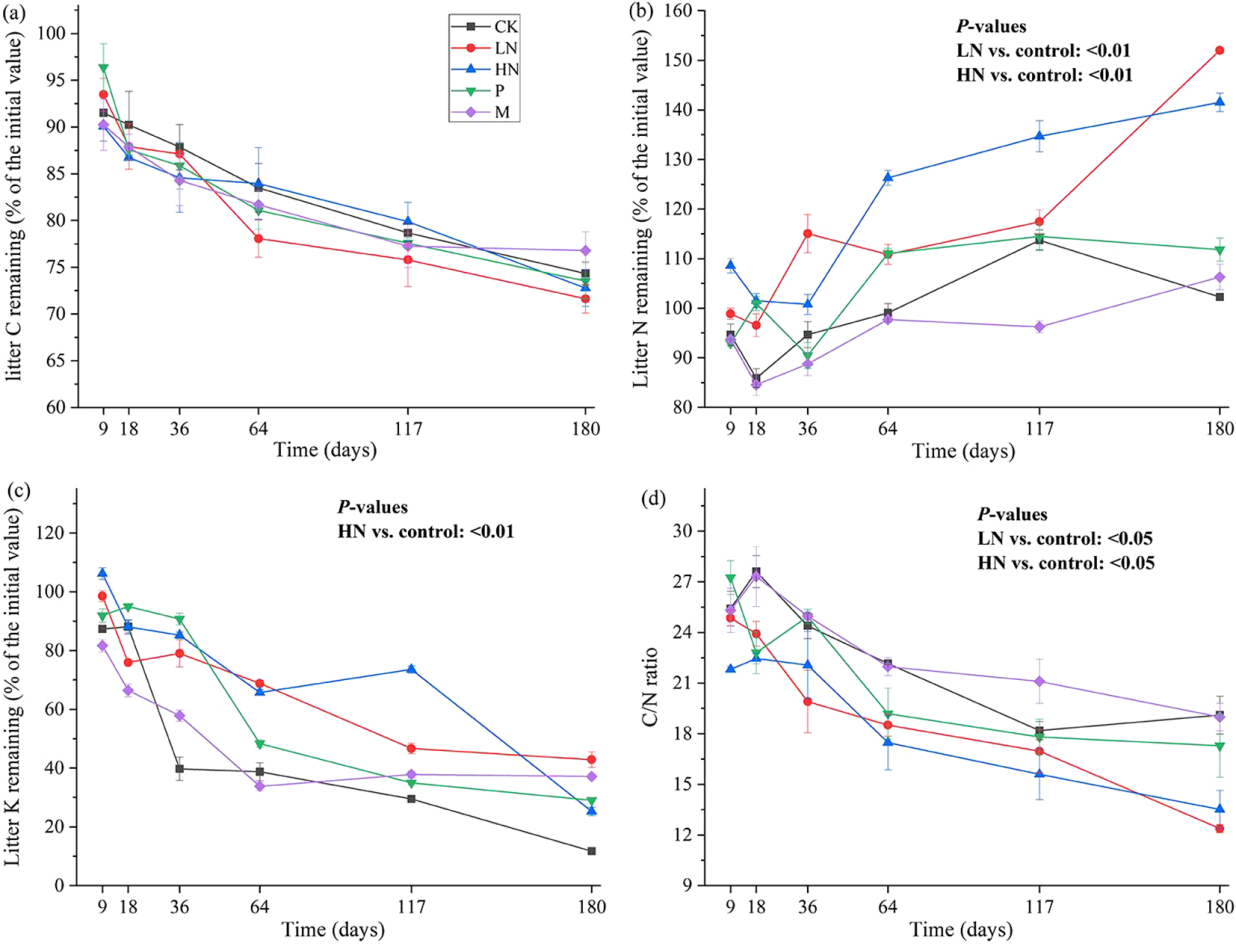
Dynamics of residual C **(A)**, N **(B)**, K **(C)**, C/N ratio **(D)** throughout the litter decomposition. CK, control; HN, high concentration N addition; LN, low concentration N addition; P, P addition; M, micronutrient addition.

**Figure 3:**
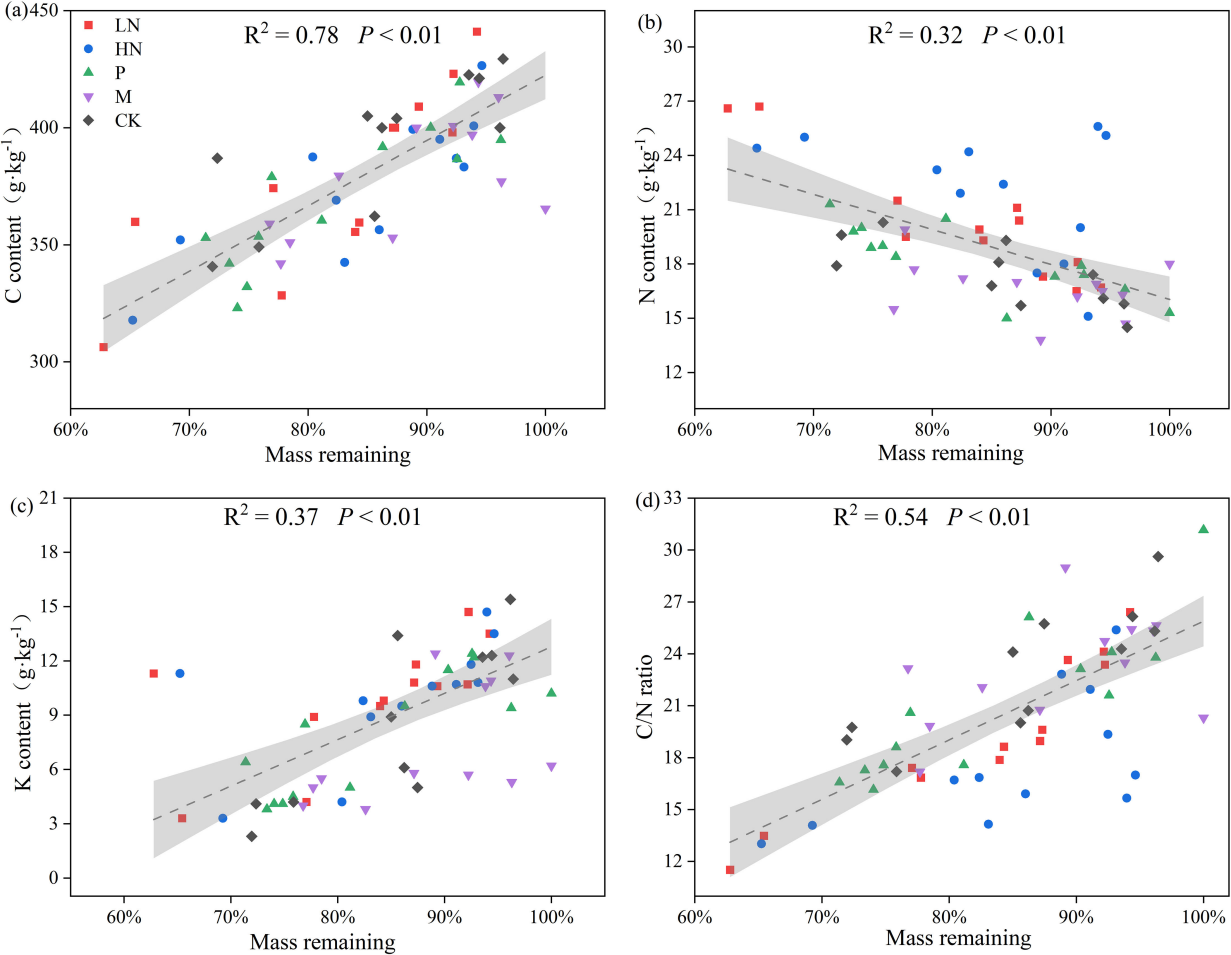
Relationships between remaining mass and C **(A)**, N **(B)**, K **(C)**, C/N ratio **(D)** in all groups. CK, control; HN, high concentration N addition; LN, low concentration N addition; P, P addition; M, micronutrient addition.

**Figure 2** shows the patterns of element release during litter decomposition. *E. japonica* litter had initial C, N, and K contents of 46%, 1.6%, and 1.4%, respectively. After 180 days of decomposition, the C content was lower than the initial value. However, there was no significant difference in the trend of C release between the fertilization group and the CK group. The trend of the N content fluctuates and eventually indicates net N accumulation. In all N-addition treatments, the average percentage of N concentrations still in the litter was 146%. During the first 18 days, the remaining groups briefly released N. Following that, they increased to approximately 100% and remained constant. The content of K decreased continuously during decomposition, and the lowest K content was 11.73% in CK group. The other groups had K levels between 25% to 42%. The N addition treatments caused a significant increase in N concentration (*p* < 0.05) compared to the other treatments, while low N addition significantly decreased K release (*p* < 0.01) (**Figure 2**). Additionally, the findings of Pearson’s analysis revealed a significantly negative correlation between the remaining mass and N content (*p* < 0.01) and a significantly positive correlation between the remaining mass with C content, K content, and C/N ratio (*p* < 0.01) (**Figure 3**).

### Impacts of nutrient addition on soil extracellular enzyme activity and microbial community

**Figure 4:**
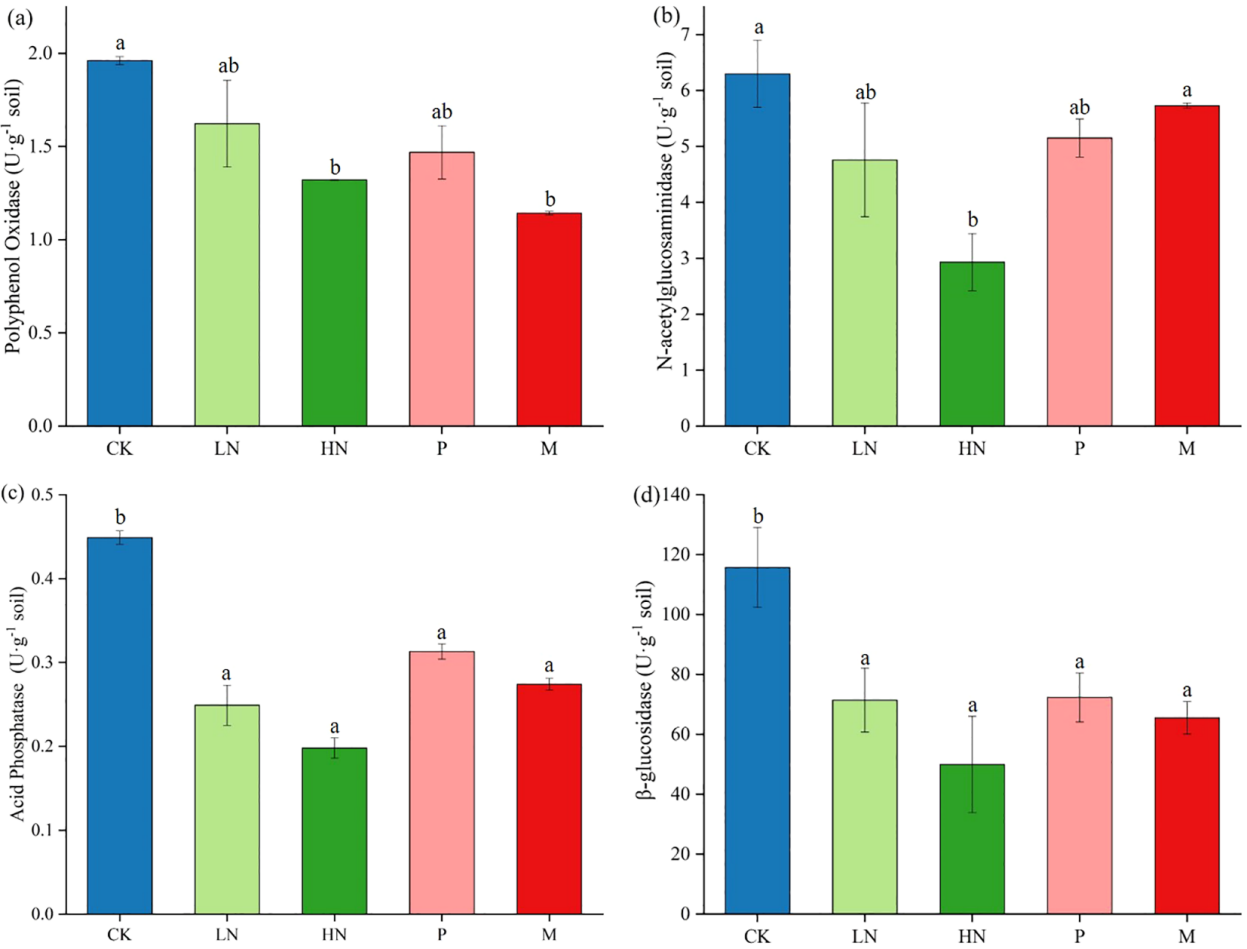
The soil enzyme activities of each treatment in the later phase of litter decomposition (**A**. Polyphenol oxidase, **B**. N-acetylglucosaminidase, **C**. Acid phosphatase, **D**. β-glucosidase). Different lowercase letters denote a significant difference between the treatment and control (*p* < 0.05). CK, control; HN, high concentration N addition; LN, low concentration N addition; P, P addition; M,micronutrient addition.

**Figure 5:**
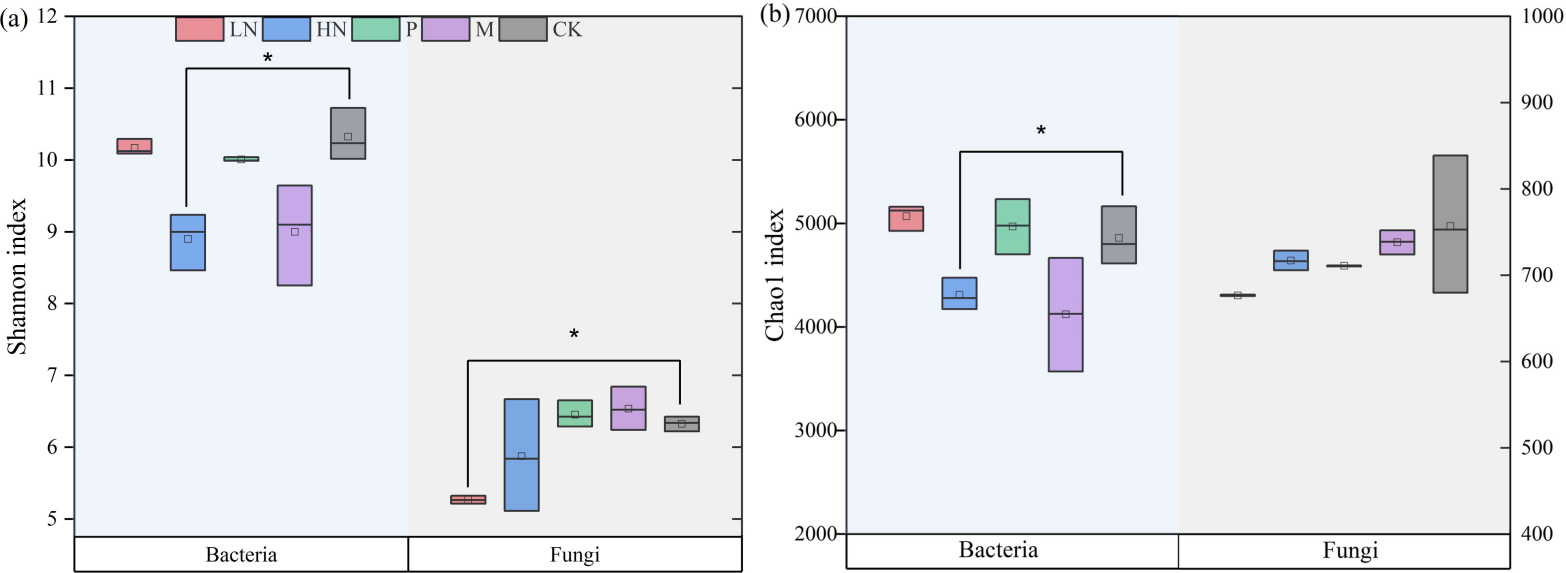
Soil microbial diversity index of each treatment in the later phase of litter decomposition (**A**. Shannon index, **B**. Chao 1 index). Asterisk (*) denotes a significant difference between the treatment and control (*p* < 0.05). CK, control; HN, high concentration N addition; LN, low concentration N addition; P, P addition; M,micronutrient addition.

After the decomposition experiment, we found that the direct impacts of additional exogenous nutrients on PO, NAG, ACP, and BG activities during this study were significant. The results showed that exogenous nutrient input decreased soil enzyme activity (**Figure 4**). The high N fertilizer significantly decreased the activity of four soil enzymes. It is important to note that the CK group’s soil enzyme activities were consistently higher than those of the treatments, including nutrient addition. As seen in **Figure 5**, the low N addition treatments did not influence the diversity of the soil bacterial community, but significantly negatively impacted fungal abundance. The addition of high N fertilization treatments significantly decreased the soil bacteria’s Shannon and Chao indices. Low N fertilization treatments significantly decreased the Shannon indices of the soil fungal functional group (*p* < 0.05) (**Figure 5**). Other treatments had no appreciable impact on the diversity of the bacterial communities.

**Figure 6:**
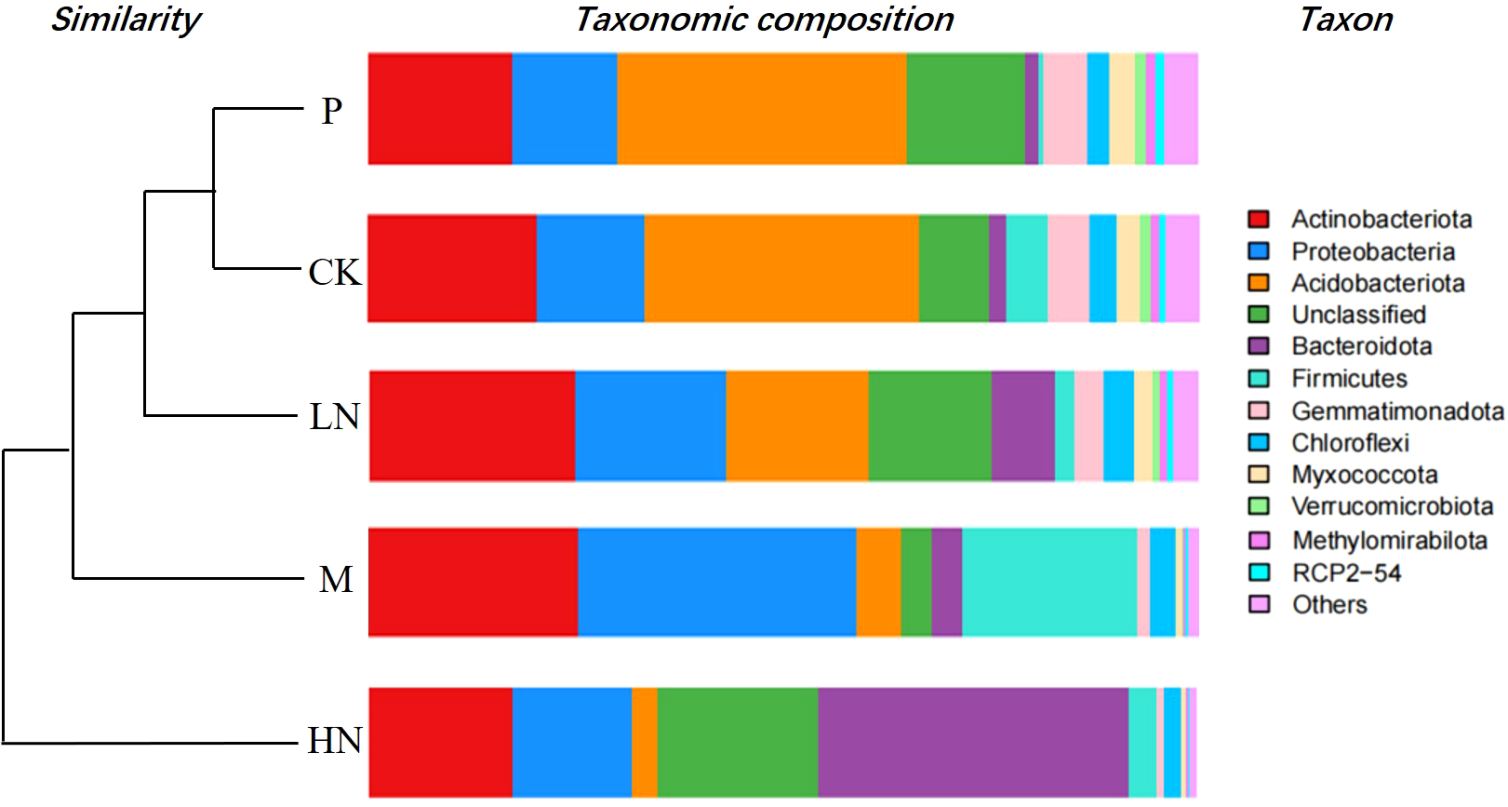
Phylum-level relative abundances of the soil bacterial community composition across all treatments. CK, control; HN, high concentration N addition; LN, low concentration N addition; P, P addition; M, micronutrient addition.

Actinobacteria, Acidobacteria, Proteobacteria, Bacteroidetes, and Firmicutes were the most prevalent bacterial phyla in all soils included in this study (**Figure 6**). The addition of nitrogen and micronutrients reduced the abundance of Acidobacteria, while HN addition increased the abundance of Actinobacteria. The addition of micronutrients increased the abundance of Proteobacteria. The soil fungal community consisted primarily of Ascomycota, Mucoromycota, Basidiomycota, and unclassified fungi (**Supplementary Figure S1**). There was little difference between the CK and P addition treatments. The relationship between enzyme activity and decomposition rates was not found to be significantly different. Microbial diversity index and breakdown rate were not significantly correlated in this study, except for bacterial OTUs (**Supplementary Figure S2**). **Supplementary Figure S3** displays the genus-level relative abundances of the soil community composition across all treatments.

## Discussion

### Changes in nutrient release and correlation with decomposition

As the most abundant element in the litter, C was often released continuously during the decomposition, as shown in many studies (Manzoni et al., 2010; Zhang et al., 2021). In contrast, N displayed a net accumulation in this study (**Figure 3**). Similarly, excessively high levels of N addition significantly increased litter N content in *Stipa aliena* Keng and *Elymus nutans* Griseb (Song et al., 2019). Liu also found N enrichment in a study on the decomposition of litter in ecological tea gardens (Liu et al., 2023). These results may imply that adding N influences N cycling, but not C cycling, in orchard ecosystems. The N enrichment in our study could be attributed to *E. japonica* leaf litter’s low initial N content, which was only 1.4%. Therefore, an increase in nitrogen content within a certain range will not lead to an increase in the total mass of litter. Decomposers typically immobilize N from the environment because litter has inadequate N, which causes decomposers to immobilize growth (Parton et al., 2007). N is released from the litter when its N content reaches a certain point (Berg and Staaf, 1981). A previous study has suggested that N release will occur in litters where the C/N ratio is between 25 and 34 (Chen et al., 2019). Additionally, the release of N from some litter may not be noticed for several years (Berg, 2000). In general, microorganisms were thought to be responsible for N enrichment. K was continuously released in this study because it was the most easily transferred element in the plant. It resided in the litter in ionic form and was released by osmosis.

Following the findings of this study, C content, K content, and C/N ratio were positively correlated with the remaining litter mass (*p* < 0.01), and N addition may influence decomposition by modifying litter element content. Low concentration N addition in this study altered the element release pattern by increasing litter N content and significantly decreasing the residual mass.

### Effects of nutrient addition on soil enzyme activity and microbial structure

Enzyme activity is a key factor influencing the litter decomposition rate (Burns et al., 2013). ACP catalyzes the hydrolysis of organic P molecules, while oxidase primarily decomposes lignin (Tien and Kirk, 1983; Marklein and Houlton, 2012). BG activity directly affects the rate of cellulose degradation (Sinsabaugh et al., 1993). In this study, the four enzyme activities significantly decreased after 180 days of nutrient application and decomposition. It has been reported that the decrease in C compounds was the reason for the decrease in oxidase activity caused by N addition (Hoyos-Santillan et al., 2018). In a study of N decomposition in mid-subtropical China, high N treatment decreased the activity of BG and ACP in the late phase of decomposition (Chen et al., 2012). In Hu’s work, N administration also suppressed several enzyme functions (Hu, 2020). Microorganisms release oxidase to decompose lignin to obtain N (Moorhead and Sinsabaugh, 2006); however, bacteria may restrict enzyme activity when environmental conditions meet the nutritional requirement (Allison and Goulden, 2017). Additionally, exogenous N induces the formation of recalcitrant aromatic compounds that lower extracellular enzyme activity and stoichiometric interactions between polyphenols and amino complexes (Fog, 1988). In line with previous studies (Chen et al., 2013), adding P and micronutrients in this study decreased enzyme activity. Studies have demonstrated that P limitation stimulates microbial mineralization of P from the environment, while P addition inhibits the effect of “microbial P mining” and thus reduces enzyme activity (Moorhead and Sinsabaugh, 2006). It has also been demonstrated that bacteria reduce their investment in the production of enzymes when there is enough P (Zhang et al., 2021). This may be the reason why the addition of phosphorus decreased enzyme activity in this study.

In this study, nutrient addition decreased the diversity of soil microorganisms. N addition may harm microbial growth by decreasing soil acidity (Sun et al., 2018). Other studies had similar findings suggesting a deleterious relationship between fungal abundance and soil N availability (Bahram et al., 2012; Yao et al., 2017). P limitation may promote “microbial P mining” which P addition suppresses (Deforest, 2019). The expression of C and N cycling genes is reduced and there is a significant decrease in microbial activity when soil nutrients are available, as microorganisms use inactive substrates first (Fierer et al., 2010). Adding nutrients can impact microbial abundance by altering soil stoichiometry and the amount of fungal secretion, and by disrupting the initial nutritional balance (Wei et al., 2013). However, while microbial turnover efficiency may be increased, the decrease in abundance does not always imply that decomposition is inhibited (Li et al., 2022).

After 180 days of decomposition, the dominant species of soil microorganisms were the same as in many previous types of research (Tian et al., 2020; Xie and Yin, 2022). Acidobacteria and Proteobacteria play significant roles in litter decomposition (Pankratov et al., 2011). Acidobacteria decompose complex macromolecules, including lignin and cellulose (Llado et al., 2016). Proteobacteria use the ammonia and methane produced during decomposition (Lv et al., 2014) to decompose simple organic matter (Wang et al., 2022). Through network analysis and subsequent culturing, Zheng revealed keystone taxa involved in the dynamics of microbial litter decomposition, including Actinobacteria, Bacteroidetes, and Chloroflexi (Zheng et al., 2021). Lu’s research has shown that Gemmatimonadetes and Firmicutes are involved in litter decomposition of *Robinia pseudoacacia* Linn. and *Quercus acutissima* Carr (Lu et al., 2022). Basidiomycota and Ascomycota are the two primary groups of fungi that decompose the cell walls of leaf litter. Basidiomycetes decompose refractory compounds in litter by producing lignin-degrading enzymes (Castro and Freitas, 2000). Ascomycetes mainly decompose cellulose and hemicellulose (Baldrian, 2017). The reason for the change in microbial abundance may be that the input nutrients altered the growth strategy of microorganisms, and it is also related to the functional adaptability of microorganisms (Xie and Yin, 2022). The microbial diversity index and soil enzyme activity had almost no significant link with this study’s decomposition rate and remaining mass (Appendix S2). The only factor that is significantly (*p* < 0.5) positively correlated with the rate of remaining mass is the number of 16S OTUs. However, it is possible that the study period was not long enough for short-term alterations to be significant.

### Mechanisms of nutrient addition affecting litter decomposition

Adding exogenous nutrients can impact litter decomposition by altering the litter element content, soil enzyme activity (Li et al., 2011), and microbial growth or activity (Li et al., 2017; Yan et al., 2018). Low N addition in this study promoted decomposition by increasing N content, which significantly negatively correlated with the remaining mass. High N addition increased N content but decreased K release, positively correlating with the remaining mass. The highest number of 16S OTUs positively correlating with the remaining mass was found in the micronutrient addition group. This may be the cause of the inhibition of decomposition caused by the addition of micronutrients.

As an economic fruit tree, *E. japonica* is widely planted in southern China. Therefore, this study provides a basis for the effects of fertilization on litter decomposition and nutrient cycling in orchards. However, the results are limited, as the study only focused on one type of litter. Furthermore, only soil enzyme activity and microorganisms in the final degradation phase were investigated in this study. The final results have limitations, and further research into the specific relationship between litter decomposition and soil microbial dynamics would require long-term monitoring of soil microbial dynamics.

## Conclusion

The main conclusions of this study are as follows: 1) low N addition decreased residual mass, whereas the addition of micronutrients increased it. 2) Our findings showed that adding N increased the N concentration in the litter. The rate of the remaining mass was highly correlated with C concentration, K concentration, and the C/N ratio. N addition may affect how quickly litter decomposes by altering the content of the elements. 3) In the late stages of decomposition, nutrient addition also decreased soil enzyme activity and microbial diversity. Our findings could be used as a reference for researchers studying the mechanism of litter decomposition in fertile soil, and forecast element cycling of orchard ecosystem in the face of fertilizer through changes in nutrients during litter decomposition.

## Data Availability

Publicly available datasets were analyzed in this study. Data are available from Figshare: https://figshare.com/articles/dataset/Nie_xlsx/22954334.
