# Supplementary material for: Effects of fertilization on litter decomposition dynamics and nutrient release in orchard systems
Source: Front Plant Sci. 2024 Dec 5;15:1467689. doi: 10.3389/fpls.2024.1467689 (PMC11655230; doi:10.3389/fpls.2024.1467689)
Supplement: Supplementary file 1 [file DataSheet1.docx]

Table S1. Initial element content in litters of *Eriobotrya japonica*.

| Element | C (g/kg) | N (g/kg) | K (g/kg) | Cellulose | Lignin |
| --- | --- | --- | --- | --- | --- |
| Content | 464 | 16.5 | 14 | 19% | 24% |

Table S2. The chemical property of the soil

| Element | SOC (g/kg) | TN (g/kg) | TP (g/kg) |
| --- | --- | --- | --- |
| Content | 36.5 | 3.40 | 0.016 |


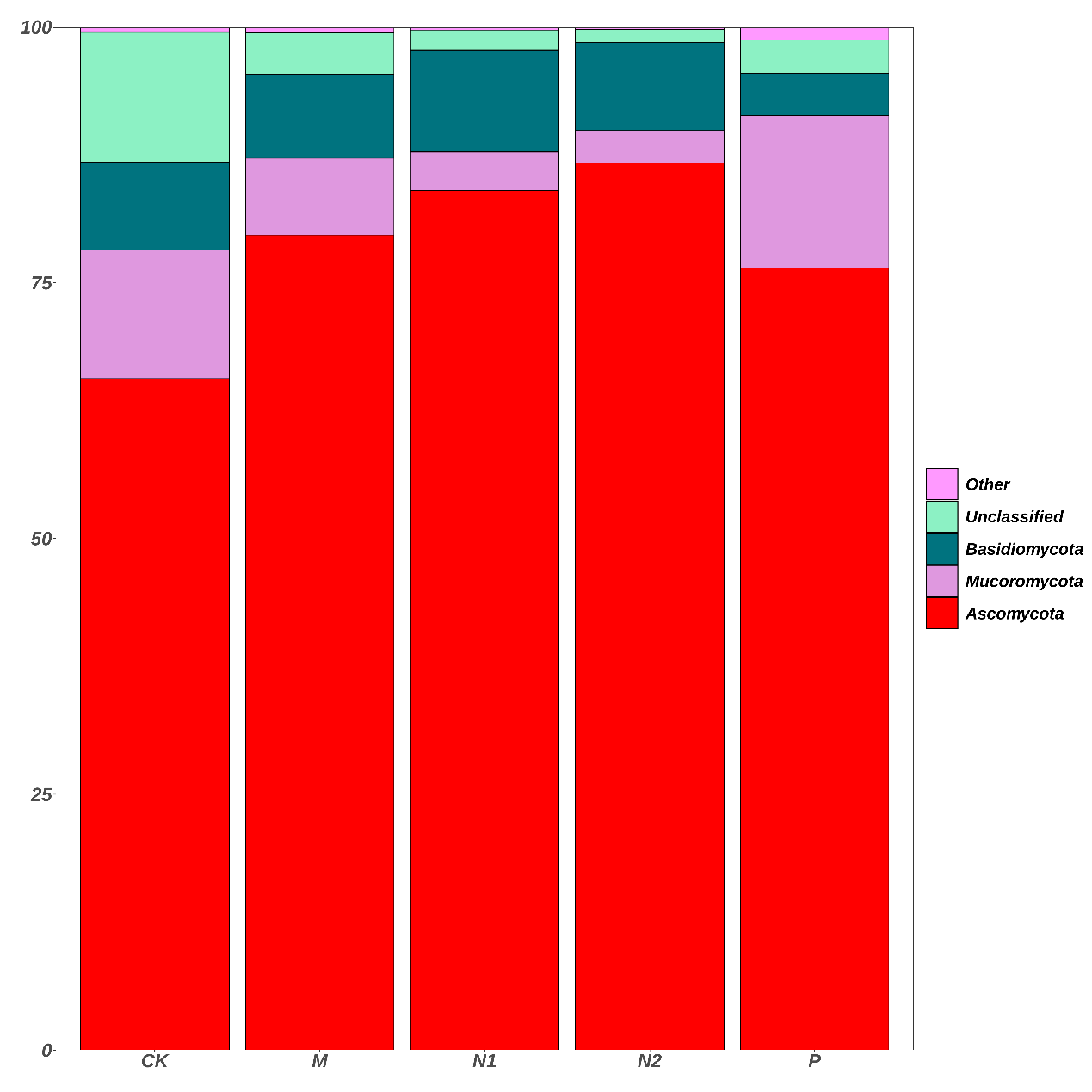


Fig. S1. Phyla-level relative abundances of soil fungal community composition across all treatments.


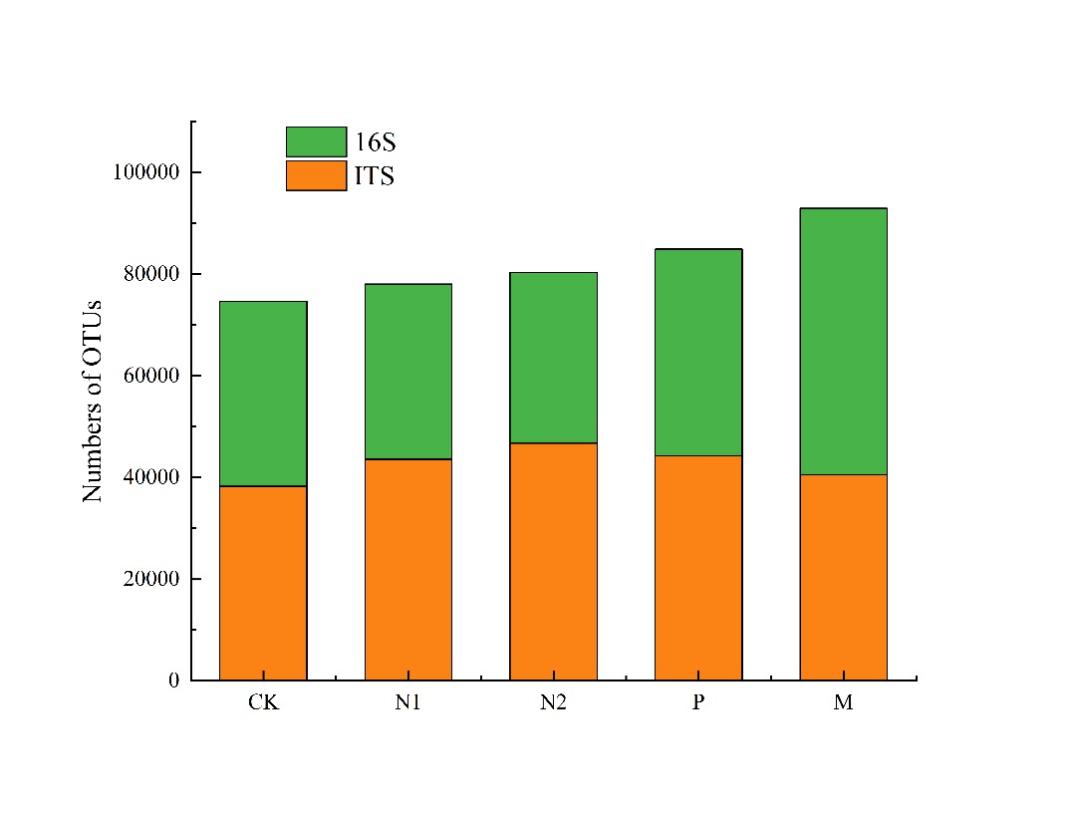


Fig.S2. Number of OTUs in different treatments in the later stage of litter decomposition


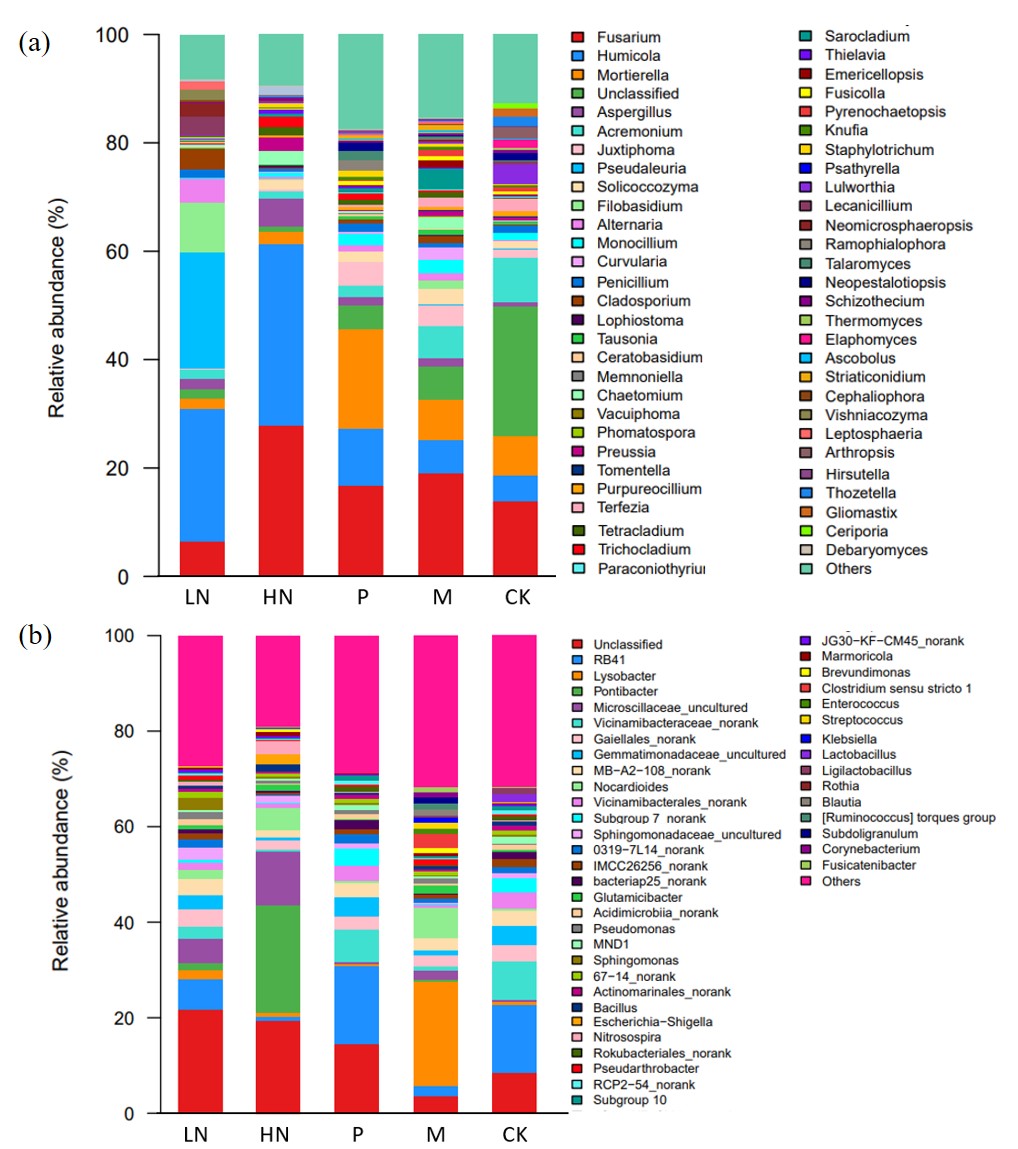


Fig. S3. Genus-level relative abundances of soil fungal (a) and bacterial (b) community composition across all treatments.
